# The Effects of Pilates in Parkinson’s Disease—A Narrative Review

**DOI:** 10.3390/life15071035

**Published:** 2025-06-29

**Authors:** Józef Alfons Opara, Jarosław Wojciech Szczygieł, Krzysztof Mehlich, Katarzyna Szczygieł

**Affiliations:** 1 Institute of Physiotherapy and Health Sciences, Jerzy Kukuczka Academy of Physical Education, ul. Mikołowska 72b, 40-065 Katowice, Poland; j.szczygiel@awf.katowice.pl (J.W.S.); k.mehlich@awf.katowice.pl (K.M.); 2 Department of Neurology, Clinical Hospital No. 1 Named After Prof. Stanisław Szyszko, ul. 3 Maja 13/15, 41-800 Zabrze, Poland; 3 Department of Neurology, Upper Silesian Medical Center Named After Prof. Leszek Giec, ul. Ziołowa 45/47, 40-635 Katowice, Poland; kbszczygiel@gmail.com

**Keywords:** body–mind interventions, balance, motor functions, Parkinson’s disease, PD, Pilates

## Abstract

Parkinson’s disease (PD) is one of the most well-known neurodegenerative diseases. Axial symptoms of PD include tremors in the arms and legs, stiffness of the muscles in the limbs and trunk, slow movement, impaired coordination, and balance disorders. Progressive disability increases the risk of falls and leads to immobilization of the patient. Comprehensive rehabilitation plays a very important role in the treatment process and serves mainly to improve motor functions and balance. In recent years, traditional methods of rehabilitation have been enriched by sometimes unconventional modern methods, which are attractive to patients. Unfortunately, current scientific evidence for the effectiveness of these methods is insufficient. Unconventional methods being used increasingly often in the rehabilitation of patients with PD include mind–body interventions. One of these interventions is Pilates exercise, which works on a physical and mental level. In this narrative review, we present the state of the art on the effects of Pilates exercise on balance and motor functions in PD. Previous studies, the results of which are available in scientific reports, have not provided convincing evidence for the effectiveness of these methods. Between 2019 and 2024, four systematic reviews and meta-analyses on the use of Pilates in the rehabilitation of patients with PD were published. Most of the reports show many shortcomings: too small groups of patients; frequent methodological errors, such as a lack of randomization and insufficient inclusion and exclusion criteria; imprecise descriptions of the interventions; different intensities and frequencies of exercises; too different outcome measures; and poorly chosen methods of statistical evaluation. Therefore, many authors emphasize the need for further, better-planned research.

## 1. Introduction

Parkinson’s disease (PD) is one of the most common neurodegenerative diseases. The number of patients with PD is expected to increase with the overall aging of the population. The disease most often appears between 50 and 60 years of age and affects over 4% of people over the age of 85; thus, it is a critical medical and social issue. Axial symptoms of PD include tremors in the arms and legs, stiffness of the muscles in the limbs and trunk, slow movement, impaired coordination, and balance disorders. People with PD require rehabilitation due to progressive motor disability manifested by gait disturbances and a tendency to fall.

Comprehensive rehabilitation plays a very important role in the treatment process, its main purpose being to improve walking and balance. Recent methods of rehabilitation of patients with PD use the phenomenon of neuroplasticity. This phenomenon is closely related to PD-specific mechanisms, especially those related to the degeneration of dopaminergic pathways and the reorganization of the cerebral cortex. Neuroplasticity refers to the ability of the brain to reorganize and adapt its structure and function in response to various stimuli. In the 21st century, traditional rehabilitation methods have been joined by newer methods that are often attractive to the patient. Currently understood PD rehabilitation is a combination of traditional therapies and innovative approaches, whose main goal is to improve motor functions, cognitive functions and the overall quality of life. Unfortunately, current scientific evidence for the effectiveness of these methods is insufficient. Unconventional methods being increasingly used in the rehabilitation of patients with PD include Pilates. Pilates exercises are mind–body interventions that work on a physical and mental level.

Most studies on Pilates for patients with PD report many shortcomings: too small groups of patients; frequent methodological errors such as lack of randomization and insufficient inclusion and exclusion criteria; imprecise description of the intervention; different intensity and frequency of exercises; too different outcome measures; and poorly chosen methods of statistical evaluation.

## 2. Exercise Following Pilates

Pilates is a type of mind–body exercise developed in the early 20th century by German exercise trainer Joseph Pilates, who called his method “Contrology”. It is practiced worldwide, particularly in developed countries. Pilates uses a combination of about 50 repetitive exercises, each stemming from the “five essentials”: breathing, neck alignment, rib and shoulder blade stabilization, pelvic mobility, and use of the transverse abdominis muscle. Each exercise is typically repeated three to five times [1]. Pilates is constantly evolving, and exercises can be performed on both a mat and on specialized equipment, such as spring-based resistance machines called “reformers.” In mat Pilates, people sit or lie down, using their body weight as the primary resistance, using gravity to stabilize the spine.

There are five main types of Pilates: (1) Classic Pilates: from the first techniques created by Joseph Pilates; (2) Yoga Pilates: a blend of Pilates and yoga; (3) Stott Pilates: gentle; (4) Power Pilates: for muscular and spiritual strengthening; and (5) Reform Pilates: carrying out exercises with a specific machine made up of several accessories [2]. Wells et al. conducted a systematic review of the Pilates literature and compared the definitions used in studies involving healthy participants and those with low back pain. Ultimately, 119 papers met the inclusion criteria; in 41% of the articles (*n* = 49), the participants were healthy, while in 14% of the articles (*n* = 17), the participants had low back pain. The remaining articles (*n* = 53) focused on people with other pathologies. The results suggest that Pilates is a mind–body exercise that focuses on strength, core stability, flexibility, muscle control, posture, and breathing. Exercises can be performed on a mat or involve the use of specialized equipment. Posture was discussed significantly more often in studies involving participants with low back pain (LBP) than in studies involving healthy participants. Evidence from LBP studies should be contextualized for PD (e.g., core stability as a transdiagnostic mechanism). The authors conclude that there is general consensus in the literature on the definition of Pilates exercises. More emphasis may be placed on posture for people with LBP, while traditional principles, other than breathing, may be less important [3].

In 2015, Professor Chris Baggoley prepared a published report commissioned by the Australian Department of Health reviewing the existing literature on 17 alternative therapies, including Pilates, to determine whether any of them were suitable for coverage by health insurance. The experts employed concluded that, due to the small number and methodologically limited nature of existing studies, the effectiveness of Pilates was uncertain [4]. Byrnes et al. published, in 2018, a systematic literature review on the effectiveness of Pilates as a rehabilitation tool in a wide range of conditions in an adult population. Inclusion criteria were met by 23 studies published between 2005 and 2016, most of which were of high quality according to the CONSORT and PEDro scales. These articles assessed the effectiveness of Pilates in the rehabilitation of low back pain, ankylosing spondylitis, multiple sclerosis, postmenopausal osteoporosis, nonstructural scoliosis, hypertension, and chronic neck pain. A total of 19 studies showed that Pilates was more effective compared to a control or comparison group in improving pain and disability [5].

Patti et al. (2024) [6] found 1566 papers on the effectiveness of Pilates for LBP, of which 36 were included in a systematic review and 19 in a meta-analysis. A total of 22 studies compared the effects of Pilates with those of no exercise, and 13 studies examined the effects of Pilates with those of non-specific exercise. The analysis showed that Pilates had a positive effect on the perception of LBP compared with no exercise and non-specific exercise [6].

## 3. Comprehensive Rehabilitation in PD

Comprehensive rehabilitation is an important element of treatment in PD. The latest methods of rehabilitation for PD patients use the phenomenon of neuroplasticity. Khan et al., in a review article on neurorehabilitation, referred to the definition of Cramer et al.: “Neuroplasticity is the ability of the nervous system to respond to internal and external stimuli by reorganizing its structure, functions, and connections” [7,8]. According to Braun and Bock (2011), the brain is a self-organizing system adapted to a specific environment before and after birth [9]. Self-organization was defined in 2009 by Prokopienko as the evolution of the system into an organized form in the absence of external pressure [10]. Khan et al. found ample evidence of stimulation of neuroregeneration processes in response to multiple rehabilitation interventions [7].

The effectiveness of rehabilitation in PD was assessed by Tomlinson et al. in 2013 [11], who presented the results of a systematic review of the Cochrane database: a total of 33 studies with 1518 participants was identified. Compared with no intervention, physical therapy significantly improved walking speed, distance traveled during a two- or six-minute walk test, stride length, functional mobility and balance, reach test range, and disability. There was no difference in falls and quality of life as assessed by the patients. Comparing the different types of physiotherapy interventions showed no evidence of any superiority of one type over the others [11].

Ramaswamy et al. indicated in 2021 [12] that the complexity of motor and non-motor symptoms seen in PD, with their variability and progressive nature, has a significant and potentially detrimental effect on mobility and subsequent quality of life for those with the condition. A considerable body of evidence now exists advocating the positive effect of physical activity and exercise on both the motor and non-motor symptoms of PD, as well as the mitigated impact and effects of the secondary complications. The literature emphasizes early adoption of higher-intensity exercise, with reported benefits at a neurophysiological level and a potential diminution in the rate of progression of the condition [12].

Animal studies and a small number of human-based studies have demonstrated that high-intensity exercise promotes improved vascularization through angiogenesis, as well as increased concentrations of neurotrophic factors such as BDNF or GDNF, which are essential for neuronal growth and integrity [13].

Jones et al. stated in 2022 that participation in moderate- to high-intensity exercise (65–85% of mHR) has been shown to be both safe and feasible for people with Parkinson’s disease and is associated with potential neurorestorative effects [14]. Many authors have reported evidence that varied exercise styles that generate strength and power through resistance training may be beneficial for motor symptoms in PD. These benefits are associated with better balance, gait parameters, functional ability, and quality of life. Further improvement is reported where early rehabilitation combines coordination of limb and trunk movements and increasing challenges to cognitive ability, through dual- and multiple-task exercise routines. Exercise could also be beneficial for non-motor symptoms associated with improved sleep, fatigue, and mood, as well as reductions in constipation, depression, anxiety, and apathy. Several authors have reported a connection between exercise participation, enhanced memory, and associated executive functions. They noted a positive impact on cognitive abilities, including rational thinking (planning and organizing), reading, learning, and memory. Reduced discomfort from musculoskeletal and central pain and other medical conditions affecting general health and mobility and slowing down of disease progression have also been observed after [14]. In a study proposing a wheel as a tool to help plan exercise for people with PD, Julie Jones et al. stated that yoga, Pilates and stretching improve flexibility and should be practiced 2–3 times a week [14].

These have been several reports on treadmill training with virtual reality (VR) for improving gait performance and reducing fall risk in PD. However, there is no consensus on the optimal training duration [14].

Brandín-De la Cruz et al. (2020) [15] reported the results of a pilot study involving nine patients with PD. The intervention consisted of 12 sessions, each session lasting 30 min, regularly spaced over four consecutive weeks. Participants trained on a treadmill with a partial weight support of 20% of their body weight and were equipped with a virtual reality helmet controlled by a two-handed joystick. There were significant differences in the small-to-average effect sizes when comparing the before and after values of walking distance, walking speed, balance, and quality of life, confirming the feasibility of combining an anti-gravity treadmill and an immersive VR system for rehabilitating PD patients [15].

In 2020, Radder et al. [16] published a meta-analysis of 191 studies with 7998 participants. Conventional physical therapy has been shown to significantly improve motor symptoms, gait, and quality of life in PD patients, while resistance training improves gait. Treadmill training improves gait. Strategic training (including dual task) improves balance and gait. Video games improve balance and quality of life. Dancing, Nordic walking, balance and gait training, and martial arts improve motor symptoms, balance, and gait, while hydrotherapy has a beneficial effect on balance. Tai Chi and Qigong, referred to as martial arts, were used in 11 studies (*n* = 580). Martial arts interventions were also compared with no exercise or sham treatment and showed beneficial effects on motor symptoms, balance and gait parameters. Martial arts had a moderate effect on TUG and a moderate effect on (MDS)-UPDRS, gait speed and step length [16].

In 2022, Carignano et al. [17] published a Cochrane systematic review of the effects of robot-assisted gait training (RAGT) on gait in patients with PD. The final 20 of 230 results were included: two systematic reviews, nine randomized controlled trials, four uncontrolled studies, and five narrative reports. A total of nine studies used an exoskeleton device, while the remaining studies used a robot with an end effector. There was a large variability in disease-related disability. In conclusion, despite the association with white matter hyperdensity (WMH) volume, RAGT showed benefits in gait, and no adverse events were recorded. However, this training does not seem superior to other interventions, except in patients with more severe symptoms and advanced disease [17].

In 2022, Pelosin et al. suggested that longer treadmill training and VR training lead to greater improvement in cognitive functions, especially in those directly affected by the virtual environment, based on a clinical study of 77 patients with PD stages II-III according to Hoehn and Yahr, aged 40 to 70 years [18]. Bukowska et al. proved the effectiveness of neurologic music therapy training for mobility and stability in PD [19].

In 2023, Qian et al. [20] compared the effectiveness of 24 types of exercises in the treatment of postural instability in adults with PD through a systematic review and network meta-analysis. The exercises were aerobic exercise, aquatic exercise, balance and gait training, balance and gait training with external cue or attention, body weight support treadmill training, classic physiotherapy program, dual-task multicomponent exercise, multidisciplinary exercise, robot-assisted gait training, resistance training, Tai Chi, treadmill training, VR, and whole body vibration. This review focused on different balance outcome categories: balance test batteries, static steady-state balance, dynamic steady-state balance, proactive balance, and reactive balance. A total of 199 studies with 9523 patients were eligible for qualitative synthesis. The best results were found for treadmill training with partial body weight support (ttPBWS), aquatic exercise, and Pilates. Balance and gait training with external cue or attention (BGT_ECA) and robot-assisted balance and gait training (RA_GT) also had good effects, but the level of evidence was often low. The authors concluded that there is low-quality evidence that ttBWS, aquatic exercise (AQE), Pilates, BGT_ECA, and RA_GT are possibly the most effective treatments, pending outcome of interest, for adults with PD [20].

Ernst et al. (2024) [21] published a systematic review and network meta-analysis of exercise for people with PD. They included 154 RCTs with a total of 7837 participants, mostly with mild to moderate disease and without severe cognitive impairment. The number of participants in each study was small (mean 51, range 10–474). They found evidence for beneficial effects of most types of exercise on motor symptoms and quality of life (QoL) in PD patients, but little evidence for differences between these interventions. Clearly, this review emphasizes the importance of exercise for the primary outcomes of motor symptom severity and QoL, whereas the specific type of exercise may be secondary. Although the evidence for the effect of exercise on the risk of adverse events is very equivocal, the interventions included in this review were described as relatively safe. Finally, the authors concluded that larger, well-conducted studies are needed to increase confidence in the evidence [21].

In 2024, Palm et al. [22] conducted a systematic review and meta-analysis of RCTs that examined the effects of group exercise versus individual exercise on motor function and mobility in people with PD. A total of 23 studies met the inclusion criteria and were included in the quantitative analysis. There was no significant difference in motor function and mobility between group and individual exercise in all standardized outcome meta-analyses. Motor function and mobility were significantly improved with group exercise versus individual exercise in 9 of 11 standardized outcome meta-analyses. Based on low- to moderate-quality evidence, the researchers concluded that group exercise has similar to greater effects on improving motor function and mobility in people with PD [22].

Zhao et al. used a network meta-analysis approach to quantify the information from pooled RCTs on motor interventions that improve motor function in patients with PD. Interventions included were treadmill training, strategic training, aerobic exercise, aquatic exercise, balance and gait training, dual-task training, boxing, dance (e.g., Irish dancing, Sardinian dancing, square dancing, couple dancing, various rhythmic dance therapies), Qigong fitness (e.g., Eight Pieces of Brocade, Five Animal Play, Six Word Formula), Tai Chi, mindfulness and meditation, resistance exercise (e.g., weightlifting, resistance band exercises, progressive resistance exercise), high-, medium-, and low-intensity exercise, sports games, and rock climbing. In order to compare and rank exercises that could effectively improve the motor functions of patients with PD, indices such as MDS-UPDRS-III, TUG, BBS, Mini-BES Test, 6MWT were used. Results: The network meta-analysis included a total of 111 studies with 5358 participants, 133 intervention experiments and 31 intervention measures. Although most exercise interventions showed effectiveness, the cumulative ranking curves below the surface (SUCRA) showed that archery exercises significantly improved patients’ scores on the MDS-UPDRS-III scale, significantly better than routine care. High-intensity and agility exercises (high strength and agility), referred to as high-intensity exercise or agility training or a combination of both, collectively referred to as high-intensity agility training, significantly improved the patients’ time to complete the TUG test. Dance and Tai Chi exercises significantly improved the patients’ balance ability, measured by MBES in the case of the dance intervention and BBS in the case of the Tai Chi intervention. Dance also significantly improved the patients’ walking ability in the 6MWT [23]. It must be noted that Pilates was not mentioned even once in the abovementioned 17 studies on comprehensive motor rehabilitation in PD.

## 4. Pilates in PD

### 4.1. Methods of Narrative Review

We aimed to identify all available English-language randomized controlled trials (RCTs) that evaluated the effects of Pilates on motor functions, especially gait, balance, and posture, for patients with PD. A search was performed in PubMed, EBSCO, Embase, Cochrane, Web of Science, and Science Direct databases, with searches conducted from inception to 31 December 2024. We included RCTs that used a standardized outcome assessment to identify changes in mobility, i.e., Berg Balance Scale (BBS), Forward Reach Test, Falls Efficacy Scale, 5-Times Sit-to-Stand test, preferred gait speed, fast gait speed, Mini Balance Evaluation Systems Test (Mini-BES), 6-min walk test (6MWT), 10 Meter Timed Walking Measure (10TWM), Timed “Up and Go” Test (TUG), Unified Parkinson’s Disease Rating Scale (UPDRS) for activities of daily living, and UPDRS motor (MDS-UPDRS-III) [24]. We used medical subject headings (MeSHs) and full texts related to Parkinson’s disease (PD), idiopathic Parkinson’s disease, body–mind interventions, balance, posture, motor functions, and Pilates. The exclusion criteria were as follows: (1) studies involving participants with other neurological diseases than idiopathic PD; (2) studies with incomplete data or unable to obtain statistical analysis; (3) studies using outcome measurements other than MDS-UPDRS part III, TUG, 6MWT, BBS, Mini-BES Test, e.g., original UPDRS or parts 1, 2, or 4; (4) studies without control groups or involving only single acute training protocols, abstracts, or conference poster presentations; (5) studies from non-randomized controlled trials; and (6) studies on cognitive functions in PD.

### 4.2. Results

A total of 560 articles were found, among which 31 were suitable for meta-analysis. There are many reports on exercises according to Pilates for PD, but they represent mostly low-level exercise, especially referring to long-term efficacy. It is obvious that, because of very different doses, various methods used (classic, yoga Pilates, Stott, power, and reform), and different outcome measures, standardization of Pilates protocols has currently been unsuccessful. In 2013, in a pilot study involving 10 patients (without a control group), Johnson et al. found improvement in gait, balance, and stabilization after 6 weeks (twice a week) of Pilates exercises [25]. Moon et al., in a retrospective case–control study, enrolled 30 instructors, including 10 Pilates instructors, 10 resistance exercise instructors, and 10 controls. All of them performed four different stabilization exercises, during which superficial muscle activity was measured (using EMG), while deep muscle thickness was measured using ultrasound imaging. During the four stabilization exercises, the thickness of the transverse abdominis (TrA) was significantly greater in the Pilates group than in the other two groups. The thickness of the internal oblique (IO) was significantly greater in the Pilates and resistance exercise groups than in the control group [26].

In 2017, Pandya et al. [27] presented the effect of a Pilates training program, as the intervention added to conventional methods, on balance in participants with idiopathic PD. A total of 30 people with idiopathic PD aged <65 years were divided into two groups. Both groups received conventional therapy, during 21 therapy sessions of 60 min each, 3 days a week for 7 consecutive weeks. Group A was treated with conventional physiotherapy, and group B was treated with Pilates exercises with conventional physiotherapy. After 7 weeks, a statistically significant difference (<0.05) was identified between the groups in terms of functional balance, self-confidence level, and functional activities, assessed using the BBS, ABC, and TUG scales. A highly significant improvement was observed in the experimental group compared to the control group. The authors concluded that Pilates intervention with conventional balance training is more effective than conventional balance training alone in improving functional balance, self-confidence level, and functional activities in participants with idiopathic PD [27].

In 2014, Pata et al. [28] conducted a quasi-experimental study involving 35 healthy adults (61–87 years old) in an 8-week Pilates-based exercise program. A total of 32 (91.4%) participants completed post-test measures. Significant improvements were seen in the Timed Up and Go Test (TUG) (*p* < 0.001) and Turn 180 Test (*p* = 0.002). Improvements were also demonstrated in the Forward Reach Test (*p* = 0.049). A positive perception of the Pilates program and decreased fear of falling were seen. Results suggest a Pilates-based exercise program may be effective in improving balance, mobility, and postural stability to decrease fall risk [28].

Mollinedo-Cardalda et al. (2018) [29] investigated the effect of a mat Pilates (MP) exercise program with TheraBand^®^ on dynamic balance in 13 intervention and 13 control PD patients. The interventions lasted 12 weeks and consisted of two weekly sessions of 60 min each. Compared with the control group (usual gymnastics), the MP program led to greater improvements in dynamic balance and increased strength in the lower limbs, but these improvements were not sustained [29].

In 2021, Çoban et al. [30] evaluated the effects of clinical Pilates training on balance and postural control in patients with PD. Forty patients were randomly assigned to one of two groups: a clinical Pilates group (CLP) or conventional physiotherapy group (COP). Exercises were performed twice a week for eight weeks. Measurements of balance, lower limb strength, risk of falls and functional mobility showed significant increase in both groups (*p* < 0.05). In the CLP group, compared to the COP group, a significantly greater improvement in dynamic balance values was found (*p* < 0.05). Based on this, the authors concluded that CLP is as effective as COP, but with better results in dynamic balance, and thanks to this, it can be used in the rehabilitation of patients with Parkinson’s disease [30].

Mustafaoglu et al. in 2022 [31], conducted a systematic review to determine which type of MBE is most effective in improving functional ability and quality of life in patients with PD. The relative effectiveness of MBE (yoga, Tai Chi, Pilates, Qigong, and dance) in improving functional ability and disease-related quality of life (HRQoL) in patients with PD was analyzed. This review included 60 RCTs with 2037 participants. It resulted in a ranking of MBE in terms of the modification of different aspects of functional ability and health-related quality of life. No significant differences were found between Pilates, Qigong, and yoga regarding effects on functional ability. Pairwise network meta-analysis (NMA) showed that Pilates was most effective in improving functional mobility and balance performance, and yoga and dance were most effective in improving motor function, while Qigong was most effective in improving gait speed. Dance was the most effective in improving HRQoL. Based on these findings, the authors concluded that MBE should be considered as an effective strategy for improving functional ability and HRQoL in patients with PD. The most effective MBE intervention varied depending on the functional domain. Dancing was found to be an effective exercise in improving HRQoL in people with PD [31].

In 2023, Cardalda et al. [32] randomized 32 participants with PD to a low-intensity mat Pilates group (LMPG) (*n* = 16) or a high-intensity mat Pilates group (HMPG) (*n* = 16), with interventions lasting 12 weeks with two 60-min sessions per week. In LMPG, one session was performed in a standing and sitting position, and the other session was performed on the floor. HMPG participants participated in a mat-based Pilates exercise program adapted for the PD population, using a medium-resistance TheraBand^®^ (1.8 kg at 100% stretch) and 0.5 kg weighted ankle and/or wrist straps. Lower extremity strength (30-Second Chair Stand Test), gait speed (Timed Up and Go), and feasibility were assessed. Results: A total of 29 participants attended 80% of the intervention sessions. After the intervention, there was a significant increase in lower limb strength (low-intensity, 8.31% vs. high-intensity, 34.25%) and walking speed (low-intensity, 12.12% vs. high-intensity, 19.35%) [32].

Banks et al. (2024) [33] described the short-term effects of Pilates-based exercise on upper limb strength and motor coordination in people with PD. A total of 15 patients, including 4 women and 11 men, aged 66 ± 9 years, participated in a quasi-experimental clinical study. Patients underwent a 6-week (30 min/day, 3 days/week) Pilates exercise program using the Reformer, Cadillac, Chair, and Barrel equipment. Outcomes were assessed using the Nine-Hole Peg Test (9HPT), the Unified Parkinson’s Disease Rating Scale (UPDRS), bradykinesia score, and the Test D’évaluation des Membres Supérieurs des Personnes Âgées (TEMPA). The authors concluded that a short-term Pilates-based exercise program for the treatment of upper limb strength, manual dexterity, bradykinesia, and functionality is feasible and safe for people with PD. The changes in upper limb bradykinesia warrant randomized clinical trials [33].

Recently, Çoban et al. (2025) [34] presented the results of a motor learning-based RCT on clinical Pilates training for PD. A total of 32 patients in stages 2–3 of the Hoehn and Yahr scale were randomly assigned to a Parkinson Pilates group (PP) or a conventional physiotherapy group (CP). Both groups received 60-min training, twice a week, and a home physiotherapy program four times a week for 12 weeks. Gait and balance, reaction time, functional mobility, static and dynamic balance showed significant improvement in PP compared with CP, and motor examination was similar (*p* > 0.05). Functional mobility was similar (*p* > 0.05), and cadence (*p* < 0.05) showed significant improvement in PP. In addition, PP showed significant improvement in rhythm and reaction time compared with CP. These results were maintained after a 3-month follow-up [34].

During the period from 2019 to 2024, four systematic reviews on Pilates in PD have been published: Barker et al., 2015; Suárez-Iglesias et al., 2019; Sampaio et al., 2023; and de Campos Júnior et al., 2024 [35,36,37,38]. Barker et al. [35] included six studies that were suitable for meta-analysis: Bird et al., 2012; Gildenhuys et al., 2013; Irez et al., 2011; Kovach et al., 2013; Mokhtari et al., 2013; and Siqueira Rodrigues et al., 2010 [35]. Sample sizes ranged from 30 to 60 subjects, mainly women. For outcome measures, the authors used the Functional Reach Test, 4-Square Step Test, 5-Times Sit-to-Stand test, Medical Sports Performance 300, Timed Up and Go Test; the risk of falls was also taken into account. The duration of one session was 60 min; in five studies, there were three sessions per week, with two sessions in one study. The intervention took place over 5 to 24 weeks. The meta-analysis identified that Pilates can have a positive effect on balance in older adults; the number of falls was also shown to be lower after a Pilates intervention, although this was concluded from only one RCT [35].

Suárez-Iglesias et al. (2019) [36] published a systematic review and meta-analysis of the benefits of Pilates in PD. Four randomized controlled trials (RCTs) and four non-RCTs were identified. The methodological quality of the studies was poor to fair. An analysis of eight studies concluded that Pilates has beneficial effects on dexterity, balance, and functional autonomy. A meta-analysis of four RCTs found that Pilates is more effective in improving lower limb function than traditional exercise programs. Pilates can be safely recommended for people with mild to moderate PD. Preliminary evidence suggests that its use may have positive effects on dexterity, balance, and physical function. The beneficial effects of Pilates on lower body function appear to be greater than those of other conventional exercises. Based on this review, the authors concluded that future RCTs with larger numbers of patients are needed [36]. Table 1 presents characteristics of the studies included in the systematic review and meta-analysis by Suarez et al. in Medicina (Kaunas) 2019, modified.

A systematic review and meta-analysis published in 2023 by Sampaio et al. underscored the potential of Pilates training as a valuable intervention to enhance balance in the elderly population [37]. The authors finally included 20 studies that met the criteria and aims of this systematic review; of these, 11 studies were included for meta-analysis. These were Barker et al. (2016), Bird et al. (2012), Campos de Oliveira et al. (2015), Carrasco-Poyotos et al. (2019), Długosz-Boś et al. (2021), Da Silva et al. (2022), Gabizon et al. (2016), Irez et al. (2011), Josephs et al. (2016), Lima et al. (2021), Mesquita et al. (2015), Roller et al. (2018), and Siqueira Rodrigues et al. (2010) after [37]. The summary of data from these works showed that the intervention groups consisted of 10 to 44 patients, one session lasted 60 min (45 min in two studies), the frequency of sessions ranged from one to three per week, mainly two, and the duration of interventions ranged from 4 to 18 weeks, resulting in a total of 12 to 36 sessions (mainly 24 sessions). Concerning static balance, four of the eight studies included in the meta-analysis revealed significant and positive effects of the Pilates training. Dynamic balance also exhibited notable and positive improvements in six out of the nine studies that examined this [37].

Based on a meta-analysis, de Campos Jr. et al. concluded in 2024 [38] that Pilates can be recommended to improve static and dynamic postural balance in older people, but not to reduce the number of falls or fear of falling. A total of 14 studies were included, which showed satisfactory methodological quality. One session usually lasted from 45 to 60 min, the frequency of sessions ranged from two to three per week, and the duration of the intervention ranged from 4 to 24 weeks. Considering the results, there was no evidence that Pilates is superior to other forms of exercise. The certainty of the evidence ranged from very low to moderate. The authors indicated that future meta-analysis studies may show changes in the certainty and estimate of effect [38].

## 5. Summary

Using a narrative review of the literature, we attempted to define the role of Pilates exercises in the comprehensive rehabilitation of people with PD. There are many reports on this subject in the scientific literature, but they differ in quality. Pilates, a type of body–mind exercise (BME), has grown in popularity over the last fifty years, especially in developed countries, for the treatment and prevention of musculoskeletal diseases, mainly LBP. This exercise can be practiced in five ways, depending on the patient’s needs and capabilities. Between 2019 and 2024, four systematic reviews and meta-analyses on Pilates in PD have been published. In the reviewed studies, we found that there were small study groups of patients, a lack of randomization, insufficient inclusion and exclusion criteria, an imprecise description of the intervention, different intensity and frequency of exercises, very different outcome measures, and poorly chosen methods of statistical evaluation. One session was usually 60 min in duration, and 45 min in a few cases; the frequency of sessions ranged from one to three per week (mainly two), and the duration of interventions ranged from 4 to 24 weeks, which resulted in a total of 12 to 36 sessions (mainly 24 sessions). There was no evidence from the outcomes that Pilates is superior to other forms of exercise. Undesirable effects of Pilates on PD were described. Taking all of the above into account, one can conclude that there is moderate proof of the effectiveness of Pilates in improving balance, gait, postural stability, and preventing falls. Pilates can be safely recommended for patients with mild to moderate PD, i.e., in stages 1–3 of the Hoehn and Yahr scale. It is also worth adding that this intervention is safe and works on a physical and mental level.

This narrative review authorizes us to make a statement that can be seen at the end of many review papers: there is a need for further, better-planned research based on sophisticated protocols.

## Figures and Tables

**Table 1:** Summary of the studies included in the systematic review and meta-analysis by Suarez et al. in Medicina (Kaunas), published by MDPI, 2019, modified (Data from [36]).

Author(s)/ Year	Study Design	Sample Size	Intervention	Outcome Measure	Results
Daneshmandi et al., 2017	RCT	IG = 15 CG = 15	60 min, 24 sessions	TUG, FAB	improvement in functional balance and falling risk in IG
Johnson et al., 2013	single subject	IG = 10	60 min, 12 sessions	TUG, BBS, ABC, 5 min walk, posturography	improvement in BBS and 5 min walk
Hartmann et al., 2014	single- subject	IG = 7	60 min, 20 sessions	30SCS, TUG, CSR, PDQ, turnaround time, 30 s EF, one-legged stance test	Improvement in 30SCS, CSR, 30 s EF
Bakhshayesh et al., 2017	RCT	IG = 15 CG = 15	60 min, 24 sessions	30SCS, FAB, S-D test, TFE, TEE, TLFE	Improvement in all tests, nonsignificant
Pandya et al., 2017	RCT	IG = 15 CG = 15	60 min, 21 sessions	TUG, BBS, ABC	Improvement in all tests, significant
Do Carmo et al., 2018	single- subject	IG = 4	60 min, 30 sessions	30SCS, CSR, Back-Scratch, 2-Minute Step, 8-Foot Up and Go test, Arm Curl	Improvement in CSR, Back-Scratch, 8-Foot Up and Go, and Arm Curl, nonsignificant
Cancela et al., 2018	single- subject	IG = 16	60 min, 24 sessions	30SCS, CSR, Back-Scratch, 2-Minute Step, 8-Foot Up and Go, PDQ, AC	Improvement in 30SCS, CSR, 2-Minute Step, PDQ, AC
Mollinedo-Cardalda et al., 2018	RCT	IG = 12 CG = 10	60 min, 24 sessions	30SCS, TUG, FTSS	Improvement in all tests, significant

Note: IG—Intervention group; CG—Comparison group; 30SCS—30-Second Chair Stand Test (number of times the participant can sit down and stand up from a chair in 30 s); TUG—Timed Up and Go Test; FTSS—Five-Times Sit to Stand; FAB—Fullerton Advanced Balance Scale; S-D—Step-Down Test; TFE—Trunk Flexion Endurance Test; TEE—Trunk Extension Endurance Test; TLFE—Trunk Lateral Flexion Endurance Test; BBS—Berg Balance Scale; ABC—Activity-specific Balance Confidence Scale; CSR—Chair Sit-and-Reach Test; BS—Back-Scratch Test; 2MS—2-Minute Step Test; 8FUG—8-Foot Up and Go Test; AC—Arm Curl Test; PDQ—Parkinson’s Disease Questionnaire; TAT—Turnaround Time; 30 s EF—30-S Elbow Flexion Test; OLS—One-Legged Stance Test; SES—Schwab and England; 5MW—5 m Walk.

## References

[1] Kloubec J. (2011). Pilates: How does it work and who needs it. Muscles Ligaments Tendons J..

[2] Latey P. (2001). The Pilates method: History and philosophy. J. Bodyw. Mov. Ther..

[3] Wells C., Kolt G.S., Bialocerkowski A. (2012). Defining Pilates exercise: A systematic review. Complement. Ther. Med..

[4] Baggoley C., Chair of Natural Therapies Review Advisory Committee, Department of Health (2015). Review of the Australian Government Rebate on Natural Therapies for Private Health Insurance.

[5] Byrnes K., Wu P.J., Whillier S. (2018). Is Pilates an effective rehabilitation tool? A systematic review. J. Bodyw. Mov. Ther..

[6] Patti A., Thornton J.S., Giustino V., Drid P., Paoli A., Schulz J.M., Palma A., Bianco A. (2024). Effectiveness of Pilates exercise on low back pain: A systematic review with meta-analysis. Disabil. Rehabil..

[7] Khan F., Amatya B., Galea M.P., Gonzenbach R., Kesselring J. (2017). Neurorehabilitation: Applied neuroplasticity. J. Neurol..

[8] Cramer S.C., Sur M., Dobkin B.H., O’Brien C., Sanger T.D., Trojanowski J.Q., Rumsey J.M., Hicks R., Cameron J., Chen D. (2011). Harnessing neuroplasticity for clinical applications. Brain.

[9] Braun K., Bock J. (2011). The experience-dependent maturation of prefronto-limbic circuits and the origin of developmental psychopathology: Implications for the pathogenesis and therapy of behavioural disorders. Dev. Med. Child Neurol..

[10] Prokopenko M. (2009). Guided self-organization. HFSP J..

[11] Tomlinson C.L., Patel S., Meek C., Herd C.P., Clarke C.E., Stowe R., Shah L., Sackley C.M., Deane K.H.O., Wheatley K. (2013). Physiotherapy versus placebo or no intervention in Parkinson’s disease. Cochrane Database Syst. Rev..

[12] Ramaswamy B., Jones J., Baker K., Oliver B. (2021). Methodology of exercise resources development for professionals providing services for people with Parkinson’s: A technical report. Physiotherapy.

[13] Ellis T., Rochester L. (2018). Mobilising Parkinson’s disease: The future of exercise. J. Parkinson’s Dis..

[14] Jones J., Baker K., Ramaswamy B. (2022). Physical activity and exercise for people with Parkinson’s. Adv. Clin. Neurosci. Rehabil..

[15] Brandín-De la Cruz N., Secorro N., Calvo S., Benyoucef Y., Herrero P., Bellosta-López P. (2020). Immersive virtual reality and antigravity treadmill training for gait rehabilitation in Parkinson’s disease: A pilot and feasibility study. Rev. Neurol..

[16] Radder D.L.M., Silva de Lima A.S., Domingos J.H.J., Keus S.H.J., van Nimwegen M., Bloem B.B., de Vries N.M. (2020). Physiotherapy in Parkinson’s Disease: A Meta-Analysis of Present Treatment Modalities. Neurorehabil. Neural Repair.

[17] Carmignano S.M., Fundaro C., Bonaiuti D., Calabro R.S., Cassio A., Mazzoli D., Bizzarini E., Campanini I., Cerulli S., Chisari C. (2022). Robot-assisted gait training in patients with Parkinson’s disease: Implications for clinical practice. A systematic review. NeuroRehabilitation.

[18] Pelosin E., Ponte C., Putzolu M., Lagravinese G., Hausdorff J.M., Nieuwboer A., Ginis P., Rochester L., Alcock L., Bloem B.R. (2022). Motor-Cognitive Treadmill Training with Virtual Reality in Parkinson’s Disease: The Effect of Training Duration. Front. Aging Neurosci..

[19] Bukowska A.A., Krężałek P., Mirek E., Bujas P., Marchewka A. (2016). Neurologic Music Therapy Training for Mobility and Stability Rehabilitation with Parkinson’s Disease—A Pilot Study. Front. Hum. Neurosci..

[20] Qian Y., Fu X., Zhang H., Yang Y., Wang G. (2023). Comparative efficacy of 24 exercise types on postural instability in adults with Parkinson’s disease: A systematic review and network meta-analysis. BMC Geriatr..

[21] Ernst M., Folkerts A.K., Gollan R., Lieker E., Caro-Valenzuela J., Adams A., Cryns N., Monsef I., Dresen A., Roheger M. (2024). Physical exercise for people with Parkinson’s disease: A systematic review and network meta-analysis. Cochrane Database Syst. Rev..

[22] Palm D., Swarowsky A., Gullickson M., Shilling H., Wolden M. (2024). Effects of Group Exercise on Motor Function and Mobility for Parkinson Disease: A Systematic Review and Meta-Analysis. Phys. Ther..

[23] Zhao H., Zhang L., Yang J., Guo W., Sun C., Shi R., Wang Z. (2024). Parkinson’s disease motor intervention patterns: A network meta-analysis based on patient motor function. Front. Neurol..

[24] Opara J.A., Małecki A., Małecka E., Socha T. (2017). Motor assessment in Parkinson’s disease. Ann. Agric. Environ. Med..

[25] Johnson L., Putrino D., James I., Rodrigues J., Stell R., Thickbroom G., Mastaglia F.L. (2013). The effects of a supervised Pilates training program on balance in Parkinson’s disease. Adv. Parkinson’s Dis..

[26] Moon J.H., Hong S.M., Kim C.W., Shin Y.A. (2015). Comparison of deep and superficial abdominal muscle activity between experienced Pilates and resistance exercise instructors and controls during stabilization exercise. J. Exerc. Rehabil..

[27] Pandya S., Nagendran T., Shah A., Chandrabharuet V. (2017). Effect of pilates training program on balance in participants with idiopathic parkinson’s disease—An interventional study. Int. J. Health Sci. Res..

[28] Pata R.W., Lord K., Lamb J. (2014). The effect of Pilates based exercise on mobility, postural stability, and balance in order to decrease fall risk in older adults. J. Bodyw. Mov. Ther..

[29] Mollinedo-Cardalda I., Cancela-Carral J.M., Vila-Suárez M.H. (2018). Effect of a Mat Pilates Program with TheraBand on Dynamic Balance in Patients with Parkinson’s Disease: Feasibility Study and Randomized Controlled Trial. Rejuvenation Res..

[30] Çoban F., Kaygısız B.B., Selcuk F. (2021). Effect of clinical Pilates training on balance and postural control in patients with Parkinson’s disease: A randomized controlled trial. J. Comp. Eff. Res..

[31] Mustafaoglu R., Ahmed I., Pang M.Y.C. (2022). Which type of mind-body exercise is most effective in improving functional performance and quality of life in patients with Parkinson’s disease? A systematic review with network meta-analysis. Acta Neurol. Belg..

[32] Cardalda I.M., de Oliveira I.M., Suárez H.V., Carral J.M.C. (2023). Is high intensity Pilates exercise treatment beneficial for people with Parkinson’s disease?. Retos.

[33] Banks H.C., Lemos T., Oliveira L.A.S., Ferreira A.S. (2024). Short-term effects of Pilates-based exercise on upper limb strength and function in people with Parkinson’s disease. J. Bodyw. Mov. Ther..

[34] Çoban F., Kaygisiz B.B., Selcuk F. (2025). Motor Learning-based Clinical Pilates Training for the Parkinson’s Disease Rehabilitation @Parkinsonpilates: A Parallel Group, Randomised Controlled Trial with 3-Month Follow-up. Complement Ther Med..

[35] Barker A.L., Bird M.L., Talevski J. (2015). Effect of pilates exercise for improving balance in older adults: A systematic review with meta-analysis. Arch. Phys. Med. Rehabil..

[36] Suárez-Iglesias D., Miller K.J., Seijo-Martínez M., Ayán C. (2019). Benefits of Pilates in Parkinson’s Disease: A Systematic Review and Meta-Analysis. Medicina.

[37] Sampaio T., Encarnação S., Santos O., Narciso D., Oliveira J.P., Teixeira J.E., Forte P., Mrais J.E., Vasques C., Monteiro A.M. (2023). The Effectiveness of Pilates Training Interventions on Older Adults’ Balance: A Systematic Review and Meta-Analysis of Randomized Controlled Trials. Healthcare.

[38] de Campos Júnior J.F., de Oliveira L.C., Dos Reis A.L., de Almeida L.I.M., Branco L.V., de Oliveira R.G. (2024). Effects of Pilates exercises on postural balance and reduced risk of falls in older adults: A systematic review and meta-analysis. Complement. Ther. Clin. Pract..

